# STING Signaling Drives Production of Innate Cytokines, Generation of CD8^+^ T Cells and Enhanced Protection Against *Trypanosoma cruzi* Infection

**DOI:** 10.3389/fimmu.2021.775346

**Published:** 2022-01-14

**Authors:** Raquel de Souza Vieira, Marilda Savoia Nascimento, Isaú Henrique Noronha, José Ronnie Carvalho Vasconcelos, Luiz Alberto Benvenuti, Glen N. Barber, Niels Olsen Saraiva Câmara, Jorge Kalil, Edecio Cunha-Neto, Rafael Ribeiro Almeida

**Affiliations:** ^1^ Laboratório de Imunologia, Instituto do Coração, Faculdade de Medicina da Universidade de São Paulo, São Paulo, Brazil; ^2^ Laboratório de Vacinas Recombinantes, Departamento de Biociências, Universidade Federal de São Paulo, Santos, Brazil; ^3^ Divisão de Patologia, Instituto do Coração (INCOR), Faculdade de Medicina, Universidade de São Paulo, São Paulo, Brazil; ^4^ Department of Cell Biology, University of Miami, Miami, FL, United States; ^5^ Laboratório de Imunologia Experimental e Clínica, Departamento de Clínica Médica, Faculdade de Medicina, Universidade Federal de São Paulo, São Paulo, Brazil; ^6^ Laboratório de Imunologia de Transplantes, Departamento de Imunologia, Instituto de Ciências Biomédicas, Universidade de São Paulo, São Paulo, Brazil; ^7^ Disciplina de Imunologia Clínica e Alergia, Faculdade de Medicina da Universidade de São Paulo, São Paulo, Brazil; ^8^ Instituto de Investigação em Imunologia (III), Instituto Nacional de Ciência e Tecnologia (INCT), São Paulo, Brazil

**Keywords:** STING, IFN-β, IL-6, IL-12, CD8^+^ T cell, *Trypanosoma cruzi*

## Abstract

A variety of signaling pathways are involved in the induction of innate cytokines and CD8^+^ T cells, which are major players in protection against acute *Trypanosoma cruzi* infection. Previous data have demonstrated that a TBK-1/IRF3-dependent signaling pathway promotes IFN-β production in response to *Trypanosoma cruzi*, but the role for STING, a main interactor of these proteins, remained to be addressed. Here, we demonstrated that STING signaling is required for production of IFN-β, IL-6, and IL-12 in response to *Trypanosoma cruzi* infection and that STING absence negatively impacts activation of IRF-dependent pathways in response to the parasite. We reported no significant activation of IRF-dependent pathways and cytokine expression in RAW264.7 macrophages in response to heat-killed trypomastigotes. In addition, we showed that STING is essential for *T. cruzi* DNA-mediated induction of IFN-β, IL-6, and IL-12 gene expression in RAW264.7 macrophages. We demonstrated that STING-knockout mice have significantly higher parasitemia from days 5 to 8 of infection and higher heart parasitism at day 13 after infection. Although we observed similar heart inflammatory infiltrates at day 13 after infection, IFN-β, IL-12, CXCL9, IFN-γ, and perforin gene expression were lower in the absence of STING. We also showed an inverse correlation between parasite DNA and the expression of CXCL9, IFN-γ, and perforin genes in the hearts of infected animals at day 13 after infection. Finally, we reported that STING signaling is required for splenic IFN-β and IL-6 expression early after infection and that STING deficiency results in lower numbers of splenic parasite-specific IFN-γ and IFN-γ/perforin-producing CD8^+^ T cells, indicating a pivotal role for STING signaling in immunity to *Trypanosoma cruzi*.

## Introduction

Chagas disease is caused by the flagellate protozoan *Trypanosoma cruzi* (*T. cruzi*) and affects over 8 million people worldwide. The acute infection results in mild symptoms, which include fever and muscle pain. Most individuals evolve to a chronic asymptomatic infection with low parasitism, but 30%–40% either have or will develop cardiomyopathy, digestive megasyndromes, or both (1). While effective innate and adaptive immunity promotes parasite control, imbalanced host immune responses to persistent infection are suggested to favor inflammation and the development of chronic Chagas pathology (2, 3).

Toll-like receptors (TLRs), nucleotide-binding oligomerization domain 1 (NOD1) receptor, and NOD-, LRR- and pyrin domain-containing protein 3 (NLRP3) have been described as major contributors to innate immunity against *T. cruzi*, promoting production of cytokines and nitric oxide (NO) (4–11). Interleukin (IL)-6 and IL-12 are crucial cytokines for immune-mediated resistance to *T. cruzi*, as shown either by infection of genetically deficient mice or *in vivo* cytokine neutralization (12–15). TLR signaling may also result in interferon-β (IFN-β) production, which has been previously implicated in parasite control in dendritic cells and macrophages, in addition to increasing resistance to infection in mice (16, 17).

In terms of adaptive immunity, T helper 1 (Th1) cells figure as an important source of IFN-γ, promoting activation of infected macrophages and providing help for other effector cells against *T. cruzi* (18–20). Unlike CD4^+^ T cells, *T. cruzi*-specific CD8^+^ T cells are essential for infection control, either by promoting protection during early contact with the parasite or by limiting *T. cruzi* burden during chronic infection (21–23). While perforin-producing CD8^+^ T cells have a contradictory role against *T. cruzi*, being related to myocarditis and heart damage in chronically infected mice (24, 25), IFN-γ-producing CD8^+^ T cells have been indicated as protective in both experimental models and patients (25–28).

DNA sensing is highly conserved as a cellular mechanism of response to pathogens and can be mediated by a variety of molecules, such as Z-DNA-binding protein 1 (ZBP1), leucin-rich repeat flightless-interacting protein 1 (LRRFIP1), DEAD-box helicase 41 (DDX41), IFN-γ-inducible protein 16 (IFI16), and the cyclic-GMP-AMP synthase (cGAS), leading to activation of stimulator of IFN genes (STING) (29–33). Activation of STING leads to conformational changes that trigger its oligomerization and translocation from the endoplasmic reticulum to the Golgi apparatus (34, 35). During translocation, STING recruits and activates TANK-binding kinase 1 (TBK-1), which in turn phosphorylates the interferon-regulatory factor 3 (IRF3), enabling its dimerization and translocation to the nucleus to induce type I IFN (IFN-α and IFN-β) gene expression (36). Alternatively, STING activation results in NF-κB translocation to the nucleus, where it functions together with IRF3 and other transcription factors to induce the expression of type I IFN and inflammatory cytokines such as tumor necrosis factor α (TNF-α), IL-1β, and IL-6 (37, 38). This ability of STING in orchestrating multiple DNA sensing pathways has been implicated not only in innate immunity to multiple pathogens but also in promoting effector CD8^+^ T cells against cancer (39–41).

The STING ligand cyclic di-AMP (c-di-AMP) has been successfully used as an adjuvant to increase immunogenicity of anti-*T. cruzi* vaccines and to promote protection against infection in mice (42, 43). It has also been demonstrated that *in vitro* cGAS inhibition limits macrophage response to extracellular vesicles derived from *T. cruzi*-infected cells (44). In addition, previous data indicated that TBK-1 and IRF3 are involved in IFN-β production during *in vitro T. cruzi* infection (45). Therefore, we hypothesized that STING signaling would play an important role in mediating production of innate cytokines and generation of CD8^+^ T cells against *T. cruzi*, promoting protection against acute infection.

Here, we demonstrated that STING signaling is required for expression of IFN-β, IL-6, and IL-12 in response to *T. cruzi* infection in RAW264.7 macrophages and that STING absence negatively impacts activation of IRF-dependent pathways in response to the parasite. We reported that heat-killed trypomastigotes promoted no significant activation of IRF-dependent pathways and cytokine production in RAW264.7 macrophages. In addition, we showed that STING is essential for *T. cruzi* DNA-mediated induction of IFN-β, IL-6, and IL-12 gene expression in RAW264.7 macrophages. Our results also revealed that STING signaling promotes production of key innate cytokines and generation of parasite-specific CD8^+^ T cells in mice, contributing to better control of parasitemia and heart parasitism.

## Materials and Methods

### Cell Culture, *Trypanosoma cruzi* Infection, and Cellular Transfections

Rhesus monkey kidney epithelial cells (LLC-MK2 cells) (ATCC) were routinely cultured in high-glucose Dulbecco’s modified Eagle’s medium (DMEM), supplemented with 10% fetal bovine serum (FBS) (Thermo Fisher, Waltham, MA, USA) (DMEM10) at 37°C and 5% CO_2_. These cells were infected with the *Trypanosoma cruzi* Y stain in high-glucose DMEM, supplemented with 2% FBS (DMEM2) (Thermo Fisher) to maintain the parasite.

Supernatant of *T. cruzi*-infected LLC-MK2 cells was collected, centrifuged in a 15-ml tube (Corning, Corning, NY, USA) at 1,350×*g* for 10 min and washed twice with DMEM2. After the last centrifugation, the pellet was left in DMEM2 for 2 h at 37°C and 5% CO_2_ to allow live trypomastigotes to swim. The supernatant containing live trypomastigotes was collected and filtered in a bottle top 0.22-µm filter (Corning). The filter was washed with DMEM2 to resuspend the live trypomastigotes, which were transferred to a new 15-ml tube and incubated at 56°C for 10 min. Parasite DNA was obtained by incubating approximately 200 million heat-killed trypomastigotes in 500 µl of lysis buffer (Tris.HCl 0.1 M, pH 8.5; EDTA 5 mM, pH 8.0; NaCl 0.2 M, SDS 0.2%, and 100 µg of proteinase K in water) at 37°C and 600 rpm for 18 h, followed by precipitation with isopropanol at 8,600×*g* for 5 min, washing with ethanol at 70%, centrifugation at 8,600×*g* for 5 min, and resuspension in 25 µl of DNAse/RNAse-free water. The ratios of absorbance at 260/280 and 260/230 nm were used to assess the purity of DNA with a Nanodrop 2000 (Thermo Fisher). The parasite DNA used in transfection experiments had 260/280 and 260/230 ratios of 1.92 and 2.21, respectively.

RAW264.7-Lucia™ ISG and RAW264.7-Lucia™ ISG-STING-KO macrophages (*Invivo*Gen, Toulouse, France) were plated in 24-well plates (Corning) at a density of 10^5^ cells per well in 500 µl of DMEM2 24 h before infection, exposure to heat-killed trypomastigotes, or transfections. The cells were incubated for 16 h with 3 × 10^6^ live or heat-killed trypomastigotes per well in 300 µl of DMEM2, washed with PBS (Thermo Fisher), and incubated for additional 24 h in 300 µl of DMEM2. Supernatant was collected and total RNA extracted. Alternatively, the cells were transfected with 80 ng of parasite DNA complexed with lipofectamine 2000 (Thermo Fisher) in 300 µl of OPTIMEM (Thermo Fisher) per well, accordingly to manufacturer’s instructions. As experimental controls, the cells were transfected either with 1.0 μg/ml of c-di-GMP (*Invivo*Gen) or 0.5 μg/ml of Poly I:C (*Invivo*Gen) complexed with lipofectamine 2000 in 300 µl of OPTIMEM. Lipofectamine 2000 and OPTMEM were used in negative control wells. Supernatant was collected and total RNA extracted 24 h after transfection.

### Luciferase Activity and Cytokine Measurement

Twenty microliters of supernatant from infected, heat-killed parasite-exposed or transfected RAW264.7-Lucia™ ISG and RAW264.7-Lucia™ ISG-STING-KO macrophages were mixed with 50 μl of QUANTI-Luc™ (*Invivo*Gen) and immediately read in a Smart Line TL luminometer (Titertek Berthold, Pforzheim, Germany), with an acquisition time of 1 s for determination of luciferase activity. The detection of IFN-β, IL-6, and IL-12 cytokines in the supernatant of infected RAW264.7-Lucia™ ISG and RAW264.7-Lucia™ ISG-STING-KO macrophages was performed using the Mouse Custom ProcartaPlex kit (Thermo Fisher), accordingly to manufacturer’s instructions. The samples were read with a MagPix Luminex system (Merck Millipore, Burlington, MA, USA) and analyzed using the Milliplex Analyst software (Merck Millipore).

### Ethics Statement

The study was approved by the Ethics Committee on the Use of Animals (CEUA) of the Faculty of Medicine, University of Sao Paulo (FMUSP), under protocol number 1567/2020, and carried out in accordance with Brazilian Federal Law number 11,794 on scientific use of animals and the National Institutes of Health guide for the care and use of laboratory animals.

### Mice and Experimental Infection

Six- to 8-week-old male wild-type BALB/c, wild-type C57BL6, and STING-KO mice with a C57BL6 background were maintained at the Tropical Medicine Institute II, Faculty of Medicine, University of Sao Paulo. BALB/c and C57BL6 mice were purchased from the Faculty of Medicine, University of Sao Paulo. STING-KO mice were kindly provided by Dr. Baber and are derived from his previously described laboratory colony (46). The animals were housed in groups of up to 5 per cage in a room with controlled light and temperature (12 h light/dark cycles, 21°C ± 2°C) and free access to food and water.

The *Trypanosoma cruzi* Y strain was maintained in BALB/c mice and used to infect wild-type C57BL6 and STING-KO mice. Blood was collected from euthanized BALB/c mice at the peak of infection and centrifuged at 200×*g* for 10 min. The supernatant was collected, centrifuged at 3,800×*g*, and the pellet of parasites resuspended in RPMI1640 (Thermo Fisher). Fifty thousand trypomastigotes in 200 µl of RPMI1640 were intraperitoneally injected in each C57BL6 and STING-KO mouse. Parasitemia was monitored by counting the number of trypomastigotes in 5 µl of fresh blood collected from the tail vein as previously described (47).

### Nitrite Detection

RAW264.7-Lucia™ ISG and RAW264.7-Lucia™ ISG-STING-KO macrophages were plated and incubated with live trypomastigotes for 16 h, as previously described. The cells were washed with PBS and incubated for an additional 48 h in 300 µl of high-glucose DMEM without phenol red (Nova Biotecnologia, Ribeirao Preto, Brazil). Alternatively, splenocytes from 4-, 7-, and 13-day-infected C57BL6 and STING-KO mice were incubated for 48 h at a density of 5 × 10^5^ cells in 200 µl of high-glucose DMEM without phenol red per well. The supernatant was collected and centrifuged at 15,000×*g* for 5 min. The Nitric Oxide Assay kit (Thermo Fisher) was used accordingly to manufacturer’s instructions for total nitrate and nitrite detection with an Epoch spectrophotometer (BioTek, Winooski, VT, USA).

### Real-Time PCR

Total RNA extraction from RAW264.7 macrophages, hearts, and spleens was performed using Trizol reagent (Thermo Fisher), RNeasy Fibrous Tissue kit (Qiagen, Hilden, Germany), and RNeasy mini kit (Qiagen), respectively. Synthesis of cDNA was performed using the Superscript II Reverse Transcriptase (Thermo Fisher), accordingly to manufacturer’s instructions. Real-time PCR was performed using Power SyBr green master mix (Thermo Fisher) and a QuantStudio 12k thermocycler (Thermo Fisher) with the following parameters: 95°C for 15 min, 40 cycles of 95°C for 15 s, and 60°C for 1 min. The primer sequences were as follows: HPRT1 forward 5′-GTTGGGCTTACCTCACTGCT-3′; HPRT1 reverse 5′-GCAAAAAGCGGTCTGAGGAG-3′; IFN-β forward 5′-TGGGAGATGTCCTCAACTGC-3′; IFN-β reverse 5′-CCAGGCGTAGCTGTTGTACT-3′; IL-6 forward 5′-CCCCAATTTCCAATGCTCTCC-3′; IL-6 reverse 5′-GGATGGTCTTGGTCCTTAGCC-3′; IL-12 forward 5′-GAAGTCCAATGCAAAGGCGG-3′; IL-12 reverse 5′-GAACACATGCCCACTTGCTG-3′; TNF-α forward 5′-ATGGCCTCCCTCTCATCAGT-3′; TNF-α reverse 5′-TTTGCTACGACGTGGGCTAC-3′; CXCL9 forward 5′-CCAAGCCCCAATTGCAACAA-3′; CXCL9 reverse 5′-AGTCCGGATCTAGGCAGGTT-3′; IFN-γ forward 5′-AGCAAGGCGAAAAAGGATGC-3′; IFN-γ reverse 5′-TCATTGAATGCTTGGCGCTG-3′; PRF1 forward 5′-TGGTGGGACTTCAGCTTTCC-3′; PRF1 reverse 5′-GAAAAGGCCCAGGAGGAACA-3′.

For detection of parasite DNA in the hearts of infected animals, we extracted DNA using the FlexiGene Kit (Qiagen), accordingly to manufacturer’s instructions and used previously described primer sequences (48). Real-time quantitative PCR was performed using Power Sybr green Master mix and the Quanti Studio 3 thermocycler (Thermo Fisher). The β-actin gene was used as an endogenous control and the calculation of parasitism in the heart was based on a *T. cruzi* DNA dilution curve.

### Preparation of TSKB20 Peptide

TSBK20 peptide (ANYKFTVL-NH2) was synthesized by manual solid phase peptide synthesis on NovaSyn TGR R resin (Merck) using the Fmoc/tBu strategy. 2-(1H-Benzotriazole-1-yl)-1,1,3,3-tetramethyluronium hexafluorophosphate (HBTU) (Merck) and *N*,*N*-diisopropylethylamine (DIPEA) were used in the coupling reactions and *N*,*N*-dimethylformamide (DMF) was used as solvent. Purity (>97%) was determined by RP-HPLC (Shimadzu, Kyoto, Japan). The peptide was resuspended in dimethyl sulfoxide (DMSO) at a stock concentration of 10 mg/ml and used in immunological assays at a concentration of 10 µg/ml.

### Flow Cytometry

Spleens from uninfected and 13-day-infected C57BL6 and STING-KO mice were aseptically removed and disrupted using 70 µm Cell Strainer (Corning). Red blood cells were lysed using ACK lysis buffer (Thermo Fisher); the samples were centrifuged at 300×*g* for 5 min and splenocytes resuspended in R10 medium (RPMI-1640 supplemented with 10% FBS, 2 mM l-glutamine, 1 mM sodium pyruvate, 1% vol/vol nonessential amino acid solution, 1% vol/vol vitamin solution, 40 µg/ml of gentamicin, and 5 × 10^−5^ M 2β-mercaptoethanol, all from Thermo Fisher). Splenocytes were plated in 96-well round-bottom plates (Corning) at a density of 0.5 × 10^6^ cells in 200 µl of R10 medium and stimulated with 10 µg/ml of the *T. cruzi* H2-K^b^-restricted peptide TSKB20 in the presence of 5 µg/ml of brefeldin A (BioLegend, San Diego, CA, USA) for 14 h at 37°C and 5% CO_2_. DMSO and PMA (50 ng/ml) plus ionomycin (500 ng/ml) (Sigma, St. Louis, MO, USA) were used as negative and positive control stimuli, respectively.

After stimulation, the cells were transferred to 96-well V-bottom plates (Corning), centrifuged at 300×*g* for 5 min and stained with the monoclonal antibodies anti-CD3 APC-Cy7 (BD Biosciences, Franklin Lakes, NJ, USA), anti-CD4 PerCP (BD Biosciences), and anti-CD8 PE-Cy7 (BD Biosciences) diluted in PBS for 30 min at 4°C. The cells were washed twice with PBS and fixed with BD Cytofix/Cytoperm™, accordingly to manufacturer’s instructions. Thereafter, the cells were washed twice with BD Perm/Wash™ buffer and stained with the monoclonal antibodies anti-IFN-γ APC (BD Biosciences) and anti-Perforin PE (BioLegend) diluted in BD Perm/Wash™ buffer for 30 min at 4°C. The cells were washed twice with BD Perm/Wash™ buffer and resuspended in PBS. The samples were acquired with a FACS Canto II (BD Biosciences) cytometer and analyzed with FlowJo 10 software (BD Biosciences).

### Histological Analysis

Heart samples were fixed in a 10% buffered formalin solution, dehydrated in an increasing concentration of ethanol (Merck), and embedded in paraffin. The blocks were sectioned with a thickness of 5 μm and stained with hematoxylin-eosin (H&E). The pathologist performed blinded histological analysis and provided a score for the intensity of myocarditis, as follows: (0) absence of myocarditis: absence or minimal focal inflammatory infiltrate; (1) mild myocarditis: mild, focal, or multifocal inflammatory infiltrate, with little cardiomyocyte aggression; (2) moderate myocarditis: clear inflammatory infiltrate, predominantly multifocal with occasional diffuse areas (coalescence), with multiple foci of cardiomyocyte aggression; and (3) intense myocarditis: exuberant inflammatory infiltrate, predominantly diffuse, with multiple foci of cardiomyocyte aggression.

### Statistical Analysis

The results were analyzed using the Graph Pad Prism 8 software. We used Mann-Whitney *U* test for comparisons between 2 parameters and two-way ANOVA, Tukey’s, and Bonferroni’s tests for multiple comparisons. Pearson’s correlation coefficient was used for correlation analysis.

## Results

### STING Deficiency Negatively Impacts Activation of IRF-Dependent Pathways and Cytokine Expression in Response to *Trypanosoma cruzi* Infection

**Figure 1:**
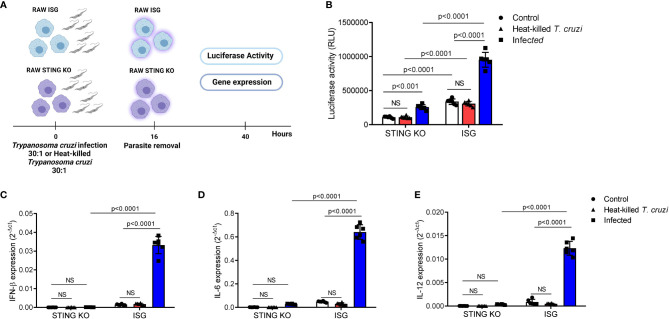
STING deficiency negatively impacts activation of IRF-dependent pathways and cytokine expression in response to *T. cruzi* infection. **(A)** Experimental procedure. **(B)** IRF-dependent luciferase activity of STING-KO and RAW264.7 ISG macrophages infected or exposed to heat-killed *T. cruzi*. **(C–E)** Real-time PCR analysis of IFN-β, IL-6, and IL-12 mRNA expression in STING-KO and RAW264.7 ISG macrophages infected or exposed to heat-killed *T. cruzi*. HPRT1 was used as housekeeping gene. Control, *T. cruzi* uninfected/unexposed STING-KO and RAW264.7 ISG macrophages; NS, no statistical significance. **(B–E)** Two-way ANOVA and Tukey’s multiple comparison test. Data are shown as mean ± SD. Experimental figure was created with BioRender.com.

Although previous data have suggested that a TBK-1/IRF3-dependent signaling pathway is essential for IFN-β induction in response to *T. cruzi* infection, the role of STING remained to be determined (45). To address this question, we used RAW264.7-Lucia™ ISG and RAW264.7-Lucia™ ISG-STING-KO macrophages, which are sufficient or deficient for STING expression, respectively, and designed to secrete luciferase into the culture medium in response to activation of IRF-dependent signaling pathways. We incubated these cells with live or heat-killed *T. cruzi* Y strain trypomastigotes for 16 h, removed residual parasites, and incubated for additional 24 h to collect supernatant and total RNA for evaluation of luciferase activity and gene expression (**Figure 1A**). We observed that although infection of STING-KO macrophages promoted activation of IRF-dependent pathways, this activation was significantly lower than that observed for RAW264.7 ISG macrophages upon *T. cruzi* infection (**Figure 1B**). On the other hand, no differences in activation of IRF-dependent pathways were found in heat-killed *T. cruzi*-exposed STING-KO or RAW264.7 ISG macrophages when compared with their respective controls (**Figure 1B**). In line with our luciferase results, we observed significantly lower IFN-β, IL-6, and IL-12 gene expression in STING-KO-infected macrophages when compared with RAW264.7 ISG-infected macrophages, while no induction of these genes was observed in either STING-KO or RAW264.7 ISG macrophages exposed to heat-killed *T. cruzi* (**Figures 1C–E**). Infection with *T. cruzi* resulted in similar induction of TNF-α gene expression in STING-KO and RAW264.7 ISG macrophages, but no significant response was observed upon exposure to heat-killed trypomastigotes (**Supplementary Figure S1A**). We also evaluated NO production in response to infection and found no difference when comparing STING-KO and RAW264.7 ISG macrophages (**Supplementary Figure S2A**). Overall, our results indicate that STING deficiency negatively impacts activation of IRF-dependent pathways and cytokine expression in response to *Trypanosoma cruzi* infection, while heat-killed trypomastigotes failed to promote activation of RAW264.7 macrophages.

### *Trypanosoma cruzi* DNA Induces STING-Dependent Cytokine Expression

**Figure 2:**
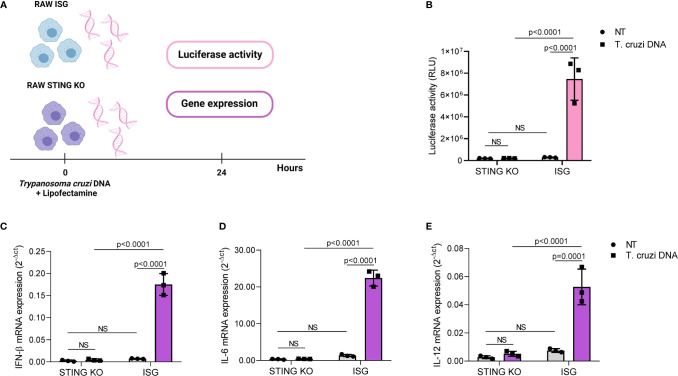
*Trypanosoma cruzi* DNA activates STING-dependent signaling. **(A)** Experimental procedure. **(B)** IRF-dependent luciferase activity of nontransfected (NT) and *T. cruzi* DNA-transfected STING-KO and RAW264.7 ISG macrophages. **(C–E)** Real-time PCR analysis of IFN-β, IL-6, and IL-12 mRNA expression in nontransfected (NT) and *T. cruzi* DNA-transfected STING-KO and RAW264.7 ISG macrophages. HPRT1 was used as housekeeping gene. NS, no statistical significance. **(B–E)** Two-way ANOVA and Tukey’s multiple comparison test. Data are shown as mean ± SD. Experimental figure was created with BioRender.com.

DNA from adenovirus 5, herpes simplex virus, *Listeria monocytogenes Plasmodium* sp., and *Leishmania donovani* activates STING-dependent signaling in a variety of cells, indicating that STING signaling may have a role in immune responses to multiple pathogens (46, 49–53). Therefore, we hypothesized that STING would be required for cytokine induction in response to *T. cruzi* DNA. To test our hypothesis, we transfected RAW264.7-Lucia™ ISG and RAW264.7-Lucia™ ISG-STING-KO cells with *T. cruzi* Y strain DNA, collected supernatant and total RNA 24 h after transfection, and evaluated luciferase activity and gene expression (**Figure 2A**). We found that STING was essential for DNA-mediated activation of IRF-dependent pathways, as STING-KO cells showed significantly lower luciferase activity upon transfection (**Figure 2B**). As observed for infection, STING-KO cells had significantly lower IFN-β, IL-6, and IL-12 gene expression in response to *T. cruzi* DNA (**Figures 2C–E**). Although STING-KO and RAW264.7 ISG cells had similar TNF-α gene expression upon infection, we found that parasite DNA transfection resulted in increased TNF-α gene expression in RAW264.7 ISG cells when compared with nontransfected control, which was not observed in STING-KO cells (**Supplementary Figure S1B**). To ensure our system was working properly, we transfected RAW264.7 ISG and STING-KO cells with poly IC (TLR3 ligand) and c-di-GMP (STING ligand). We observed that while STING-KO cells were responsive to poly IC, c-di-GMP elicited no luciferase activity or gene expression. In contrast to infection and parasite DNA, c-di-GMP failed to induce IL-6 and IL-12 gene expression while poly IC failed to induce IL-12 gene expression in RAW264.7 ISG cells (**Supplementary Figures S3A–D**). Taken together, these results indicate that *T. cruzi* DNA activates STING-dependent signaling.

### STING Signaling Increases Resistance to Acute *T. cruzi* Infection and Promotes Expression of Key Immunological Genes in the Heart of Infected Animals

**Figure 3:**
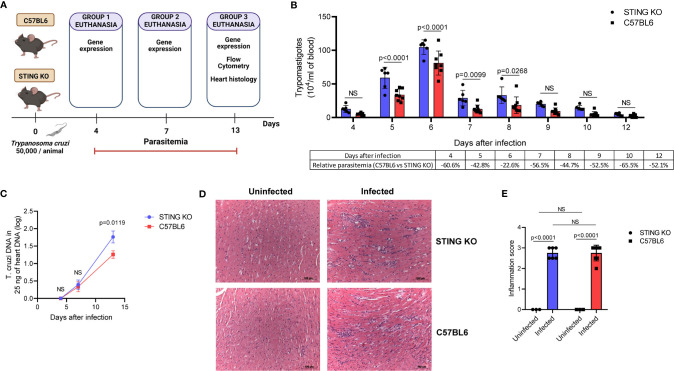
STING signaling increases resistance to *T. cruzi* infection. **(A)** Experimental procedure. **(B)** Parasitemia of STING-KO and C57BL6-infected mice. **(C)** Real-time PCR analysis of *T. cruzi* DNA in the hearts of STING-KO and C57BL6 mice 4, 7, and 13 days after infection. **(D, E)** Histological analysis of the hearts of STING-KO and C57BL6 mice 13 days after infection, ×200 magnification. NS, no statistical significance. **(B, C)** Two-way ANOVA and Bonferroni’s multiple comparison test. **(E)** Two-way ANOVA and Tukey’s multiple comparison test. **(B, D)** Data are shown as mean ± SD. **(C)** Data are shown as mean ± SEM. Experimental figure was created with BioRender.com.

Our *in vitro* results suggested that STING is required for induction of cytokines involved in *T. cruzi* immunity. Therefore, we hypothesized that STING-KO mice would have lower immune activation and would be less effective in controlling the parasite during acute infection. To test our hypothesis, we intraperitoneally infected C57BL6 and STING-KO mice (**Figure 3A**) and found that STING-KO mice had significantly higher parasitemia from days 5 to 8 after infection (**Figure 3B**). Real-time PCR analysis showed that both groups of animals had similar amounts of *T. cruzi* DNA in the heart on days 4 and 7 after infection (**Figure 3C**). However, we observed significantly higher amounts of *T. cruzi* DNA in the hearts of STING-KO-infected animals at day 13 after infection (**Figure 3C**), indicating that STING-dependent signaling plays a role in parasite control.

**Figure 4:**
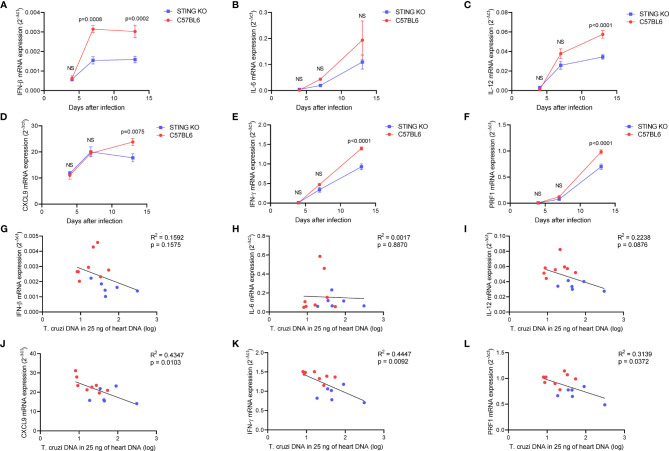
STING deficiency negatively impacts immune responses in the heart of *T. cruzi*-infected mice. **(A–F)** Real-time PCR analysis of IFN-β, IL-6, IL-12, CXCL9, IFN-γ, and PRF1 mRNA expression in the hearts of STING-KO and C57BL6 mice 4, 7, and 13 days after infection. **(G–L)** Correlation analysis of IFN-β, IL-6, IL-12, CXCL9, IFN-γ, and PRF1 mRNA expression with *T. cruzi* DNA in the hearts of STING-KO (blue circles) and C57BL6 (red circles) mice 13 days after infection. NS, no statistical significance. **(A–F)** Two-way ANOVA and Bonferroni’s multiple comparison test. **(G–L)** Pearson’s correlation. **(A–F)** Data are shown as mean ± SEM.

We performed heart histological analysis of STING-KO and C57BL6-infected animals and observed no difference in the magnitude of inflammatory infiltration 13 days after infection (**Figures 3D, E** **)**. Real-time PCR analysis of the heart tissue at days 4, 7, and 13 after infection revealed a kinetic increase in the expression of genes related to immune control of the parasite in both groups of animals. Notably, IFN-β gene expression was significantly lower in the hearts of STING-KO mice at days 7 and 13 after infection when compared with C57BL6 mice (**Figure 4A**). IL-12, CXCL9, IFN-γ, and perforin gene expression was significantly lower in the hearts of STING-KO mice 13 days after infection (**Figures 4C–F**). No significant difference in IL-6 gene expression was found in the hearts of STING-KO-infected mice when compared with C57BL6 mice (**Figure 4B**), while significantly lower TNF-α gene expression was observed in the hearts of STING-KO mice 13 days after infection (**Supplementary Figure S1C**), indicating that STING deficiency negatively impacts the expression of key genes related to protection against acute *T. cruzi* infection.

Given that all immunological genes evaluated in our study were previously shown to contribute to protection against acute *T. cruzi* infection, we performed correlation analysis to understand whether the magnitude of gene expression would be associated with parasite control. We found that IFN-β, IL-6, and IL-12 gene expression had no correlation with *T. cruzi* DNA in the heart at day 13 after infection (**Figures 4G–I**). However, CXCL9, IFN-γ, and perforin gene expression was inversely correlated with *T. cruzi* DNA (**Figures 4J–L**). Moreover, we observed a positive correlation among CXCL9, IFN-γ, and perforin gene expression in the heart of infected animals (**Supplementary Figures S4A–C**), suggesting that CXCL9-mediated recruitment of IFN-γ and perforin-expressing cells may have had a positive impact on parasite control at day 13 after infection.

### STING Signaling Promotes Expression of Innate Cytokines and Generation of CD8^+^ T Cells in the Spleen of Infected Animals

**Figure 5:**
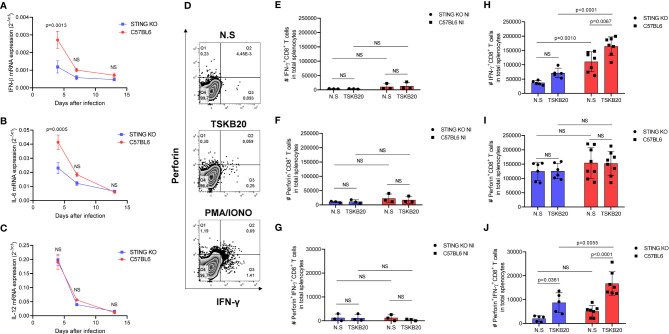
STING deficiency negatively impacts innate cytokine expression and generation of CD8^+^ T cells in response to *T. cruzi* infection. **(A–C)** Real-time PCR analysis of IFN-β, IL-6, and IL-12 mRNA expression in the spleens of STING-KO and C57BL6 mice 4, 7, and 13 days after infection. **(D)** Intracellular flow cytometry analysis of IFN-γ and perforin production by CD8^+^ T cells. **(E, F)** Number (#) of IFN-γ, perforin, and IFN-γ/perforin producing-CD8^+^ T cells in total splenocytes of uninfected mice, nonstimulated (NS) or stimulated with TSKB20 peptide 13 days after infection. **(H–J)** Number (#) of IFN-γ, perforin, and IFN-γ/perforin-producing CD8^+^ T cells in total splenocytes of *T. cruzi*-infected mice, nonstimulated (NS) or stimulated with TSKB20 peptide 13 days after infection. NS, no statistical significance. **(A–C)** Two-way ANOVA and Bonferroni’s multiple comparison test. **(E–J)** Two-way ANOVA and Tukey’s multiple comparison test. **(A–C)** Data are shown as mean ± SEM. **(E–J)** Data are shown as mean ± SD.

To have a more systemic view of the immune response to the parasite in the context of STING signaling, we evaluated the spleens of STING-KO- and C57BL6-infected mice at days 4, 7, and 13 after infection. We observed that IFN-β, IL-6 and IL-12 gene expression was higher at day 4 after infection and decreased overtime in both groups of animals (**Figures 5A–C**). Notably, STING-KO-infected mice had significantly lower IFN-β and IL-6 gene expression in the spleen at day 4 after infection when compared with C57BL6-infected mice (**Figures 5A, B** **)**, indicating that STING-dependent signaling may play a role in early induction of key cytokines against *T. cruzi*. On the other hand, IL-12 gene expression (**Figure 5C**) and TNF-α gene expression were similar in the spleens of STING-KO- and C57BL6-infected mice in all time points (**Supplementary Figure S1D**). We also evaluated NO production by splenocytes and found no significant differences between groups (**Supplementary Figure S2B**), suggesting the involvement of other signaling pathways in immunity to *T. cruzi*.

To further exploit the role of STING signaling in *T. cruzi*-driven immunity, we performed flow cytometry using splenocytes to investigate IFN-γ and perforin production by CD8^+^ T cells against a *T. cruzi* H-2K^b^-restricted peptide named TSKB20 (**Figure 5D**). As expected, we found very low numbers of splenic IFN-γ, perforin, and IFN-γ/perforin-producing CD8^+^ T cells in uninfected animals (**Figures 5E–G**). On the other hand, we observed significantly lower numbers of splenic TSKB20-specific IFN-γ and IFN-γ/perforin-producing CD8^+^ T cells in STING-KO-infected mice when compared with C57BL6-infected mice (**Figures 5H, J** **)**, while the numbers of splenic TSKB20-specific CD8^+^ T cells producing only perforin were similar in both groups (**Figure 5I**). Collectively, our results indicate that STING signaling promotes expression of innate cytokines and generation of CD8^+^ T cells against *T. cruzi*.

## Discussion

Innate and adaptive immune responses are required for controlling *T. cruzi* replication and disease establishment (54). Although TLR-dependent IFN-β production increase resistance to infection in mice (16), contrasting data have demonstrated that MyD88, TRIF, TLR-2-, TLR-3-, and TLR-4-deficient MEF, and bone marrow-derived macrophages (BMDM) still produce IFN-β in response to *T. cruzi*, while TBK-1 and IRF3 deficiency significantly impairs IFN-β production (45).

STING signaling, which is intimately related to TBK-1 and IRF3, has been studied in the context of immunity to many pathogens (39, 40). Formulations with STING ligand (c-di-AMP) as an adjuvant have been shown to increase immunogenicity of anti-*T. cruzi* vaccines (42, 43). In addition, previous work has demonstrated that *in vitro* cGAS inhibition limits macrophage response to extracellular vesicles derived from *T. cruzi*-infected cells (44). However, the role of STING during *in vitro* and *in vivo T. cruzi* infection remained to be addressed.

Here, we showed that STING is not only required for expression of IFN-β in *T. cruzi*-infected RAW264.7 macrophages but also promotes IL-6 and IL-12 expression, which are involved in host resistance to infection (12–14, 16, 17). We demonstrated that activation of IRF-dependent signaling is negatively impacted by STING absence but may also rely on other pathways, as we still observed significantly higher luciferase activity in STING-KO-infected macrophages when compared with STING-KO-uninfected macrophages. In contrast to previous data demonstrating IFN-β expression by MEF exposed to dead trypomastigotes (45), we found no significant differences in cytokine expression or luciferase activity in either STING-KO or RAW264.7 ISG cells exposed to heat-killed trypomastigotes, indicating that live trypomastigotes are required for activation of IRF-dependent pathways and cytokine expression in RAW264.7 macrophages. Whether *T. cruzi* internalization through phagocytosis occurred (55), it was not sufficient to alter our parameters in STING-KO and RAW264.7 ISG cells.

Previous data have demonstrated that adenovirus 5, herpes simplex virus, *Listeria monocytogenes*, *Plasmodium* sp., and *Leishmania donovani* DNAs activate STING-dependent signaling in a variety of cells, such as human monocytes, STING-expressing HEK293 cells, MEF, and RAW264.7 ISG macrophages (46, 49–53). Our results bring additional support to these observations by demonstrating that *T. cruzi* DNA transfection triggers robust STING-mediated activation of IRF-dependent pathways and expression of IFN-β, IL-6, and IL-12 genes, reinforcing the role of STING signaling in intracellular DNA sensing and host defense against microbial infection (39).

In line with most infections in humans, we investigated *T. cruzi*-driven immune response and parasite control using a parasite inoculum that was not lethal in acute infection. Our results demonstrated that STING absence negatively impacted parasite control, as we observed significantly higher parasitemia in STING-KO mice from days 5 to 8 of infection. Although not statistically significant, we noticed an early difference in systemic infection control, with 60% less blood parasites in C57BL6 mice at day 4 after infection, indicating that innate immunity may have had a major impact on initial parasite infection. In fact, higher IFN-β and IL-6 gene expression in the spleens of C57BL6 mice at the same period corroborates our hypothesis. Although other studies regarding innate immunity have shown distinct intensity and kinetics in parasite control, late differences in parasitemia were more frequently observed (8, 12, 14, 16). In addition, we cannot exclude that parasite strain and inoculum may have contributed to our observations.

STING deficiency resulted in significantly higher heart parasitism at day 13 after infection, suggesting impairment of local immunity. Although we found no difference in the intensity of myocardial inflammatory infiltrate, the quality of the immune response may have been affected, as suggested by lower expression of genes related to immune protection against acute infection in the hearts of STING-KO mice. The kinetics of the local immune response may also have contributed to early parasite control, as STING-KO mice presented much less-efficient IFN-β response at day 7 after infection, which was when we detected parasite DNA for the first time in the heart of infected animals. Supporting our hypothesis, previous data have demonstrated early induction of type I IFN response against *T. cruzi* Y strain at the skin of infected mice (56).

CXCL9 chemokine gene expression, known to promote migration of effector T cells to infected tissues and protective immune response against *T. cruzi* (57–60), was found to be significantly lower in the hearts of STING-KO mice at day 13 after infection, as was IFN-γ and perforin gene expression. In addition, our analysis demonstrated a positive correlation among these 3 genes, indicating that STING signaling may drive CXCL9-dependent infiltration of IFN-γ and perforin-producing cells in the hearts of acutely infected animals. Although CD4^+^ T cells have been demonstrated as an important source of IFN-γ during infection (61, 62) and natural killer (NK) cells may also migrate in response to CXCL9 (63) and express IFN-γ and perforin (64, 65), CD8^+^ T cells are still the most predominant infiltrated population in the heart (66, 67), leading to the hypothesis that CD8^+^ T cells may have played a major role in ours findings. Nevertheless, further investigation will be necessary to demonstrate whether STING signaling modulates NK and CD4^+^ T cells during infection.

Our flow cytometry analysis revealed a negative impact of STING deficiency on the numbers of splenic parasite-specific IFN-γ and IFN-γ/perforin-producing CD8^+^ T cells at day 13 after infection, which may explain why we found lower IFN-γ and perforin gene expression in the hearts of STING-KO-infected mice. In contrast to our data, TLR4-KO animals were shown to have preserved CD8^+^ T cells while having impaired innate immunity against *T. cruzi* (8), indicating a broader function of STING signaling in immune responses to the parasite. While generation of *T. cruzi*-specific CD8^+^ T cells has been shown to be unaffected by the absence of type I interferon signaling (68), we believe that impairment in the production of IFN-β, IL-6, and IL-12 against the parasite in STING-KO mice may have had a major impact on the CD8^+^ T cells. In fact, these three cytokines have been shown to promote CD8^+^ T cell activation, proliferation, and survival (69–73), supporting our hypothesis.

Perforin-producing CD8^+^ T cells have a contradictory role in acute and chronic *T. cruzi* infection, being related to myocarditis and heart damage in chronically infected mice (24, 25). In contrast, IFN-γ-producing CD8^+^ T cells have been indicated as protective in both experimental models and patients, although a dysregulated IFN-γ response may be suggested as detrimental in chronic Chagas disease cardiomyopathy (2, 25–28, 74). Here, we showed an inverse correlation between parasite DNA and the expression of CXCL9, IFN-γ and perforin in the hearts of infected animals, reinforcing a protective role for these genes in acute infection. Moreover, we found a more prominent impairment in parasite-specific IFN-γ-producing CD8^+^ T cells in STING-KO mice, suggesting that STING signaling may be responsible to promote a more effective CD8^+^ T cell-mediated immune response against *T. cruzi*. Therefore, we believe our results bring an important contribution to the field of imunoparasitology by unveiling new molecular mechanisms underlying immunity against this remarkable pathogen.

## Data Availability Statement

The original contributions presented in the study are included in the article/**Supplementary Material** Further inquiries can be directed to the corresponding author.

## Ethics Statement

The animal study was reviewed and approved by Ethics Committee on the Use of Animals (CEUA) of the Faculty of Medicine, University of Sao Paulo (FMUSP).

## Author Contributions

RV and RA contributed to conceptualization, formal analysis, methodology, investigation, writing of original draft, and manuscript revision. MN, IN, JV, and LB contributed to methodology, investigation, and formal analysis. GB contributed with resources and formal analysis. NC, JK, and EC contributed with resources and manuscript revision. EC contributed to funding acquisition. RA contributed to study supervision. All authors contributed to the article and approved the submitted version.

## Funding

This work was supported by grants from Conselho Nacional de Desenvolvimento Científico e Tecnológico to ECN (CNPq, www.cnpq.br, grant #465434/2014-2) and Fundação de Amapro à Pesquisa do Estado de São Paulo to ECN (Fapesp, www.fapesp.br, grants #2014/50890-5 and #2016/152090).

## Conflict of Interest

The authors declare that the research was conducted in the absence of any commercial or financial relationships that could be construed as a potential conflict of interest.

## Publisher’s Note

All claims expressed in this article are solely those of the authors and do not necessarily represent those of their affiliated organizations, or those of the publisher, the editors and the reviewers. Any product that may be evaluated in this article, or claim that may be made by its manufacturer, is not guaranteed or endorsed by the publisher.

## Supplementary Material

Supplementary Figure 1 STING deficiency has variable impact on TNF-α response to *T. cruzi*. **(A)** Real-time PCR analysis of TNF-α mRNA expression in STING-KO and RAW264.7 ISG macrophages infected or exposed to heat-killed *T. cruzi*. **(B)** Real-time PCR analysis of TNF-α mRNA expression in non-transfected (NT) and *T. cruzi* DNA-transfected STING-KO and RAW264.7 ISG macrophages. **(C, D)** Real-time PCR analysis of TNF-α mRNA expression in the hearts and spleens of STING-KO and C57BL6 mice 4, 7 and 13 days after infection, respectively. HPRT1 was used as housekeeping gene. NS, no statistical significance. **(A, B)** Two-way ANOVA and Tukey’s multiple comparison test. **(C, D)** Two-way ANOVA and Bonferroni’s multiple comparison test. **(A, B)** Data are shown as mean ± S.D. **(C, D)** Data are shown as mean ± S.E.M.

Supplementary Figure 2 STING deficiency has no impact on nitric oxide production against *T. cruzi*. **(A)** Nitrite detection in the supernatant of uninfected and infected STING-KO and RAW264.7 ISG macrophages 48h after infection. **(B)** Nitrite detection in the supernatant of splenocytes from STING-KO and C57BL6 mice at days 4, 7 and 13 after infection, incubated for 48h. NS, no statistical significance. **(A)** Two-way ANOVA and Tukey’s multiple comparison test. **(B)** Two-way ANOVA and Bonferroni’s multiple comparison test. **(A)** Data are shown as mean ± S.D. **(B)** Data are shown as mean ± S.E.M.

Supplementary Figure 3 STING-KO macrophages are responsive to poly IC but not to c-di-GMP transfection. **(A)** IRF-dependent luciferase activity of non-transfected (NT) and transfected STING-KO and RAW264.7 ISG macrophages. **(B–D)** Real-time PCR analysis of IFN-β, IL-6 and IL-12 mRNA expression in non-transfected (NT) and transfected STING-KO and RAW264.7 ISG macrophages. HPRT1 was used as housekeeping gene. NS = no statistical significance. **(A–D)** Two-way ANOVA and Tukey’s multiple comparison test. **(A–D)** Data are shown as mean ± S.D.

Supplementary Figure 4 CXCL9, IFN-γ and perforin gene expression positively correlates in the hearts of infected animals. **(A–C)** Pearson’s correlation analysis of CXCL9, IFN-γ and PRF1 mRNA expression in the hearts of STING-KO (blue circles) and C57BL6 (red circles) mice 13 days after infection.
