# Supplementary figures and images for: STING Signaling Drives Production of Innate Cytokines, Generation of CD8+ T Cells and Enhanced Protection Against Trypanosoma cruzi Infection
Source: Front Immunol. 2022 Jan 14;12:775346. doi: 10.3389/fimmu.2021.775346 (PMC8795786; doi:10.3389/fimmu.2021.775346)

**A**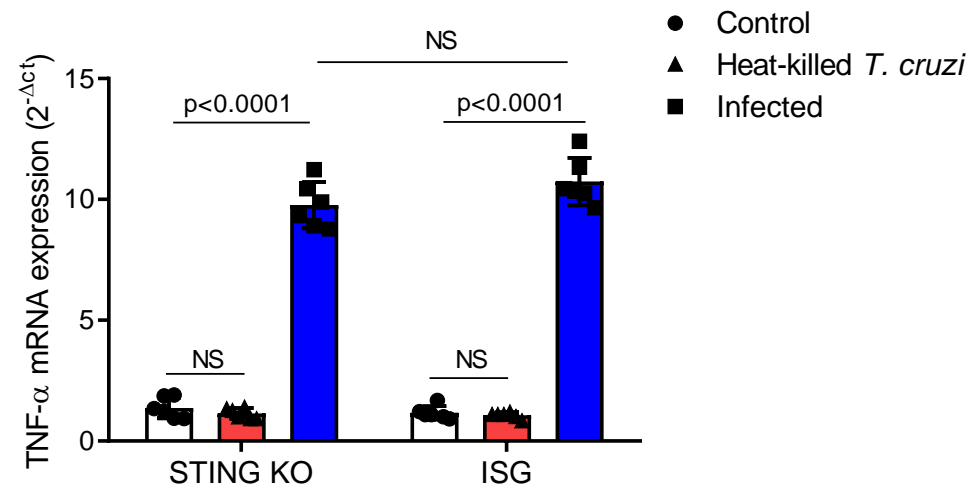**B**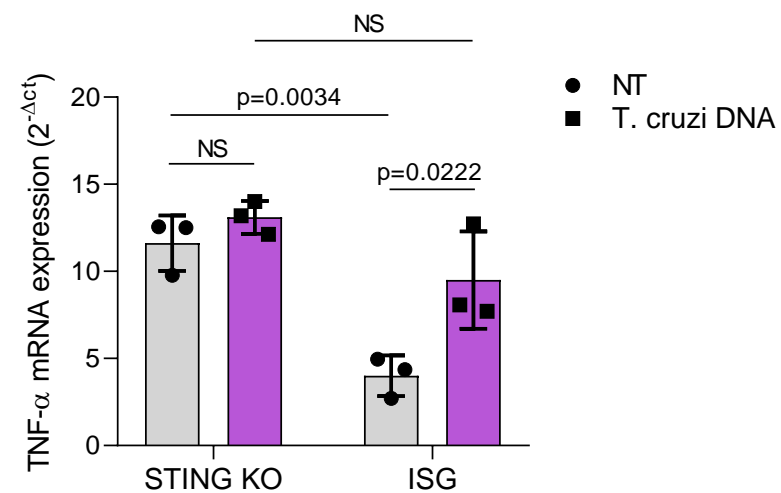**C**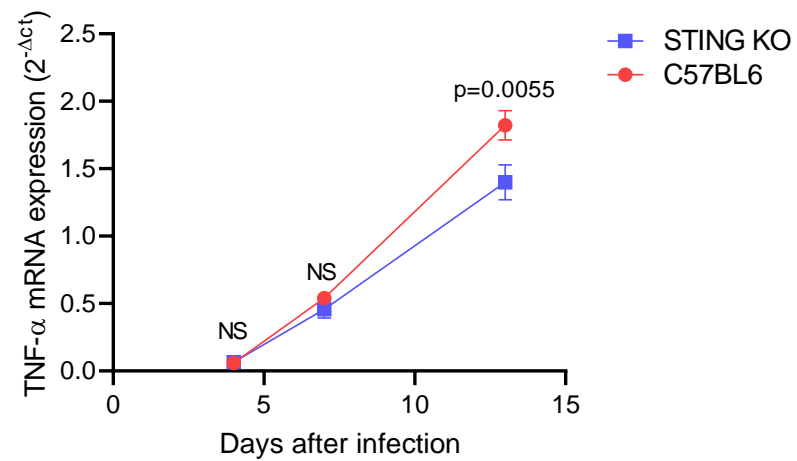**D**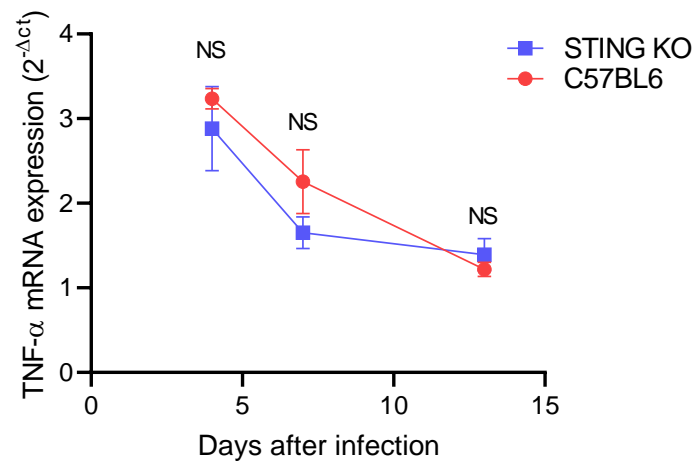

Supplement: Supplementary Figure 1 — STING deficiency has variable impact on TNF-α response to T. cruzi. (A) Real-time PCR analysis of TNF-α mRNA expression in STING-KO and RAW264.7 ISG macrophages infected or exposed to heat-killed T. cruzi. (B) Real-time PCR analysis of TNF-α mRNA expression in non-transfected (NT) and T. cruzi DNA-transfected STING-KO and RAW264.7 ISG macrophages. (C, D) Real-time PCR analysis of TNF-α mRNA expression in the hearts and spleens of STING-KO and C57BL6 mice 4, 7 and 13 days after infection, respectively. HPRT1 was used as housekeeping gene. NS, no statistical significance. (A, B) Two-way ANOVA and Tukey’s multiple comparison test. (C, D) Two-way ANOVA and Bonferroni’s multiple comparison test. (A, B) Data are shown as mean ± S.D. (C, D) Data are shown as mean ± S.E.M. [file Image_1.pdf]

**A**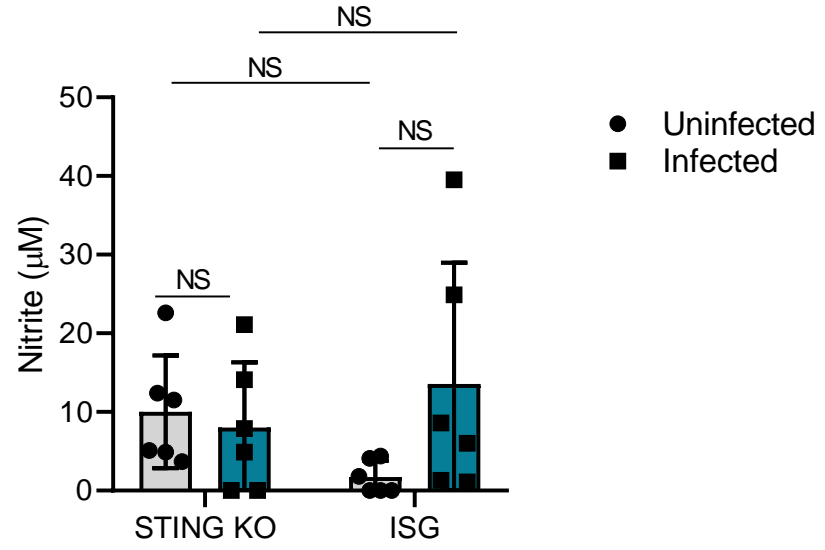**B**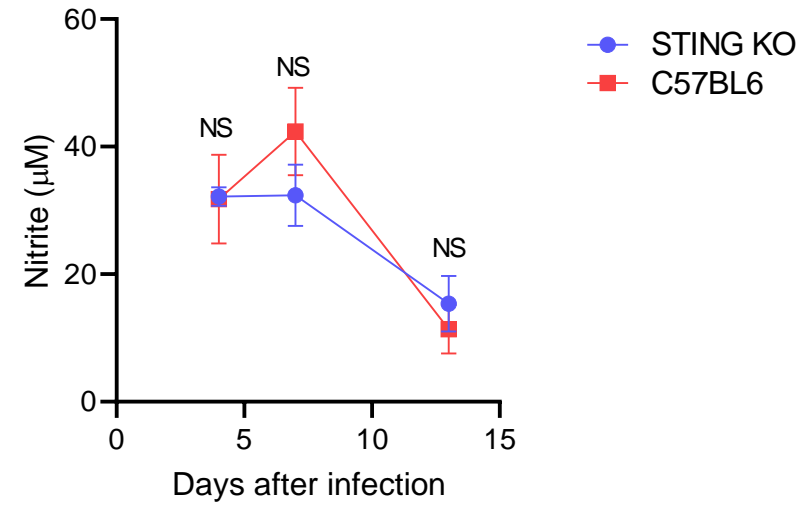

Supplement: Supplementary Figure 2 — STING deficiency has no impact on nitric oxide production against T. cruzi. (A) Nitrite detection in the supernatant of uninfected and infected STING-KO and RAW264.7 ISG macrophages 48h after infection. (B) Nitrite detection in the supernatant of splenocytes from STING-KO and C57BL6 mice at days 4, 7 and 13 after infection, incubated for 48h. NS, no statistical significance. (A) Two-way ANOVA and Tukey’s multiple comparison test. (B) Two-way ANOVA and Bonferroni’s multiple comparison test. (A) Data are shown as mean ± S.D. (B) Data are shown as mean ± S.E.M. [file Image_2.pdf]

**A**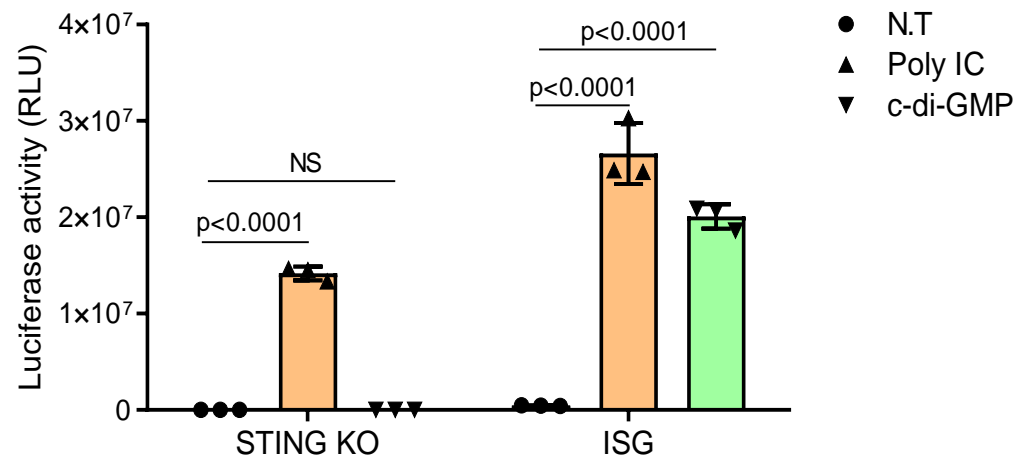**B**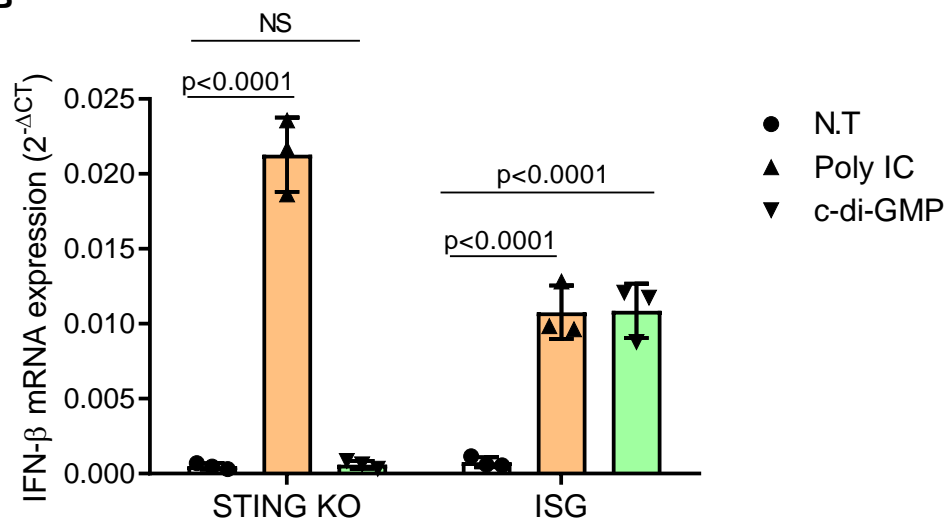**C**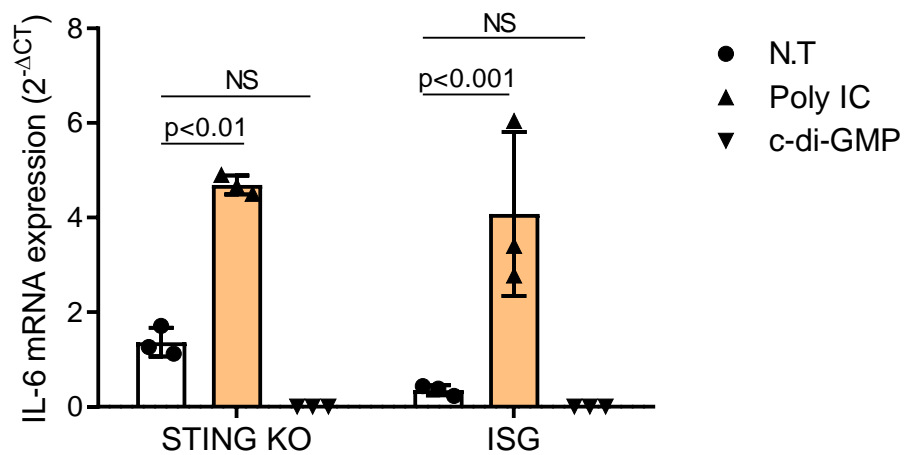**D**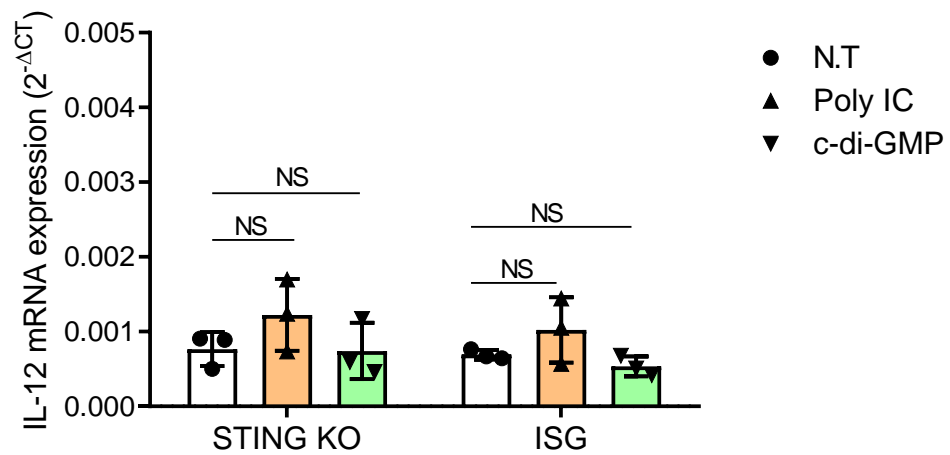

Supplement: Supplementary Figure 3 — STING-KO macrophages are responsive to poly IC but not to c-di-GMP transfection. (A) IRF-dependent luciferase activity of non-transfected (NT) and transfected STING-KO and RAW264.7 ISG macrophages. (B–D) Real-time PCR analysis of IFN-β, IL-6 and IL-12 mRNA expression in non-transfected (NT) and transfected STING-KO and RAW264.7 ISG macrophages. HPRT1 was used as housekeeping gene. NS = no statistical significance. (A–D) Two-way ANOVA and Tukey’s multiple comparison test. (A–D) Data are shown as mean ± S.D. [file Image_3.pdf]

**A**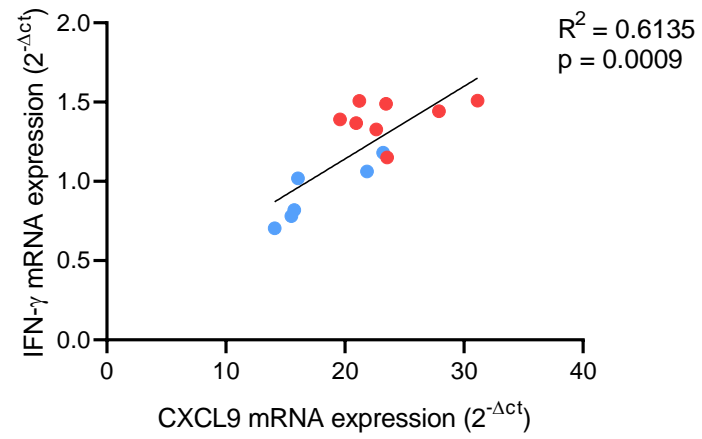**B**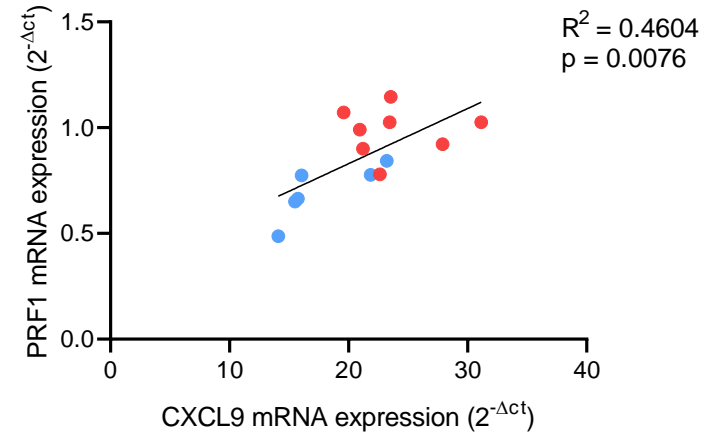**C**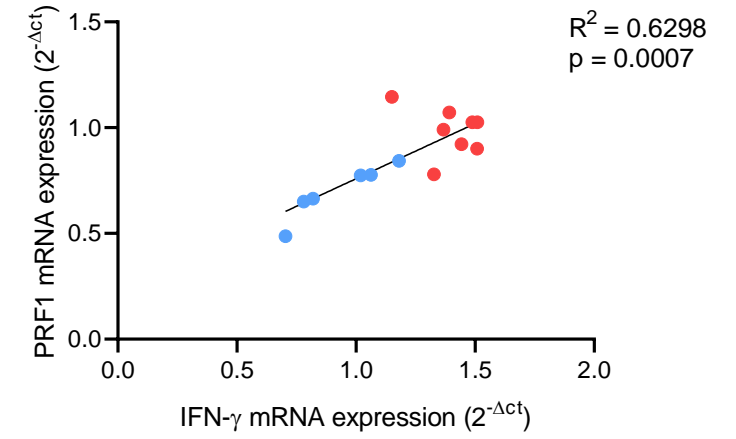

Supplement: Supplementary Figure 4 — CXCL9, IFN-γ and perforin gene expression positively correlates in the hearts of infected animals. (A–C) Pearson’s correlation analysis of CXCL9, IFN-γ and PRF1 mRNA expression in the hearts of STING-KO (blue circles) and C57BL6 (red circles) mice 13 days after infection. [file Image_4.pdf]
